# How Emotion Relates to Language and Cognition, Seen Through the Lens of Evaluative Priming Paradigms

**DOI:** 10.3389/fpsyg.2022.911068

**Published:** 2022-07-07

**Authors:** Michaela Rohr, Dirk Wentura

**Affiliations:** Cognitive Psychology Department, Saarland University, Saarbrücken, Germany

**Keywords:** evaluative priming, affect, emotion, semantic representation, valence

## Abstract

Affect and emotion are essential aspects of human life. These states or feelings signal personally relevant things or situations and color our memories and thoughts. Within the area of affective or emotion processing, evaluation–the assessment of the valence associated with a stimulus or event (i.e., its positivity or negativity)–is considered a fundamental process, representing an early and crucial stage in constructivist emotion theories. Valence evaluation is assumed to occur automatically when encountering a stimulus. But does this really apply always, even if we simply see a word? And if so, what exactly is processed or activated in memory? One approach to investigating this evaluative process uses behavioral priming paradigms and, first and foremost, the evaluative priming paradigm and its variants. In the present review, we delineate the insights gained from this paradigm about the relation of affect and emotion to cognition and language. Specifically, we reviewed the empirical evidence base with regard to this issue as well as the proposed theoretical models of valence evaluation, specifically with regard to the nature of the representations activated *via* such paradigms. It will become clear that affect and emotion are foremost (and, perhaps, even exclusively) triggered by evaluative priming paradigms in the sense that semantic affective knowledge is activated. This knowledge should be modeled as being active in working memory rather than in long-term memory as was assumed in former models. The emerging evidence concerning the processing of more specific emotion aspects gives rise to the assumption that the activation of these semantic aspects is related to their social importance. In that sense, the fast and (conditionally) automatic activation of valence and other emotion aspects in evaluative priming paradigms reveals something about affect and emotion: Valence and specific emotion aspects are so important for our daily life that encountering almost any stimulus entails the automatic activation of the associated valence and other emotion aspects in memory, when the context requires it.

## Introduction

Affect and emotion are essential aspects of human life. These states or feelings signal personally relevant things or situations and color our memories and thoughts. Within the area of affective or emotion processing, evaluation–the assessment of the valence associated with a stimulus or event (i.e., its positivity or negativity) –is considered a fundamental process, representing an early and crucial stage in constructivist emotion theories. Valence evaluation is assumed to occur automatically when encountering a stimulus. But does this really apply always even if we simply see a word? If so, what exactly is processed or activated in memory? One approach to investigating this evaluative process uses behavioral priming paradigms, and, first and foremost, the evaluative priming paradigm and its variants.

In the present review, we delineate the insights gained from this paradigm about the relation of affect and emotion to cognition and language. We will start with a theoretical outline of the concepts of emotion, affect, and evaluation to avoid misunderstandings, because researchers do not completely agree upon these concepts' definitions (see, e.g., Moors and Scherer, 2013). Of importance for the present review, this delineation shapes our view on how the processing of stimuli in evaluative priming paradigms relates to emotion and affect.

We then introduce the reader to the evaluative priming paradigm and its variants [see Herring et al. (2013) for a meta-analysis]. In the basic version of the evaluative priming paradigm, target items (e.g., words) are categorized according to their valence (Fazio et al., 1986). The target is preceded by a briefly presented prime stimulus whose valence is either congruent to the target's response category or not. Typically, faster responses and/or fewer errors are observed in cases of congruency, which is taken as an indication of automatic evaluation of the prime. We outlined the empirical evidence gathered with the paradigm, focusing on three specific questions: (1) Are “hot” emotion-related processes involved in the paradigm? Or do effects rely on the “cold” semantic categorization process (es) (or do both play a role)? We used the term “hot” to refer to all aspects related to emotion or affect that cannot be explained simply by pure semantic processing, such as, for example, the involvement of facial muscle responses, or effects relating to embodiment (e.g., modality switch costs). After having answered this question, we will address the more specific question: (2) How is valence mentally represented according to the evidence accrued with the evaluative priming paradigm? To answer this question, we describe the different task contexts under which effects in the evaluative priming paradigm have been observed (or not). It will become clear that the assessment of valence is not an integral part of prime word processing under all conditions despite its functional relevance for adaptive behavior. That is, valence assessment is not automatic in that it does not unconditionally meet all automaticity criteria (i.e., being fast, efficient, unintentional, non-conscious, and independent of current goals; Moors, 2007). It will become clear that, despite three decades of research, the existing theoretical models cannot explain all observed effects. Thus, we will propose a working memory account, integrating existing mechanisms into a coherent picture of evaluative priming. We will then ask a final question: (3) Beyond valence, can further emotion aspects—such as relevance, potency, or the specific emotion category—be involuntarily activated (and what insights may the answer to this question provide)? As the reader will see, the existing evidence is not easily explainable by (pure) semantic processing accounts so that, indeed, the evaluative priming paradigm and its variants reveal something about affect and emotion.

## What Evaluative Priming Researchers Mean When They Talk About Affect and Emotion

Affective phenomena are a very popular and widely researched topic (Dukes et al., 2021), and priming paradigms are only one approach to gain insights into them. To prevent issues akin to the parable of the blind men describing an elephant, we first provide definitions of core concepts: Affect is usually defined in a very general sense as “any experience of feeling or emotion…ranging from the simplest to the most complex sensations” (VandenBos, 2007, p. 26). Next to cognition and conation, it is identified as one of the essential components of the mind. Affect can range from a vague feeling, not bound to a specific object and, simply, phenomenally experienced to a specific emotional state that can involve cognitive aspects and can be reflected on (e.g., I experience disappointment for not having achieved a self-set goal). Often, however, researchers use the word affect in a narrower sense when referring to coarse, unspecific states that can be described as positive and negative, in contrast to specific emotional states. An emotion is seen as a temporary, dynamic process encompassing “interrelated, synchronized changes” in several components, including cognition, neurophysiology, motor expression, motivation, and subjective feeling “in response to the evaluation of an internal or external stimulus event as relevant to major concerns of the organism” (Scherer, 2005, p. 313). Important differences from affect lie in the circumscribed phenomenal feelings and related cognitions elicited by a specific stimulus or event.

For both concepts, the process of evaluation or appraisal plays a primary role, that is, the process by which an individual assigns subjective, affective meaning to stimuli. Evaluation can be broadly defined as the outcome of such a judgment process (e.g., Cunningham et al., 2007), no matter how many and which processing steps are exactly involved. However, evaluation (especially in the context of evaluative priming paradigms) is typically defined more narrowly as a mechanism that automatically evaluates all incoming stimulus information as “good/positive/pleasant” or “bad/negative/unpleasant.” More precisely, the stored mnemonic representation of the evaluative aspect of a stimulus is assumed to be activated along with its semantic representation.^1^ Whereas, the term evaluation emphasizes the cognition involved in the judgment, the term appraisal places more weight on different aspects of (and thus different kinds of information involved in) emotional processing (i.e., physiological state, expression, action tendencies, cognition, and feeling; Scherer and Moors, 2019).

This already makes clear that researchers with different backgrounds and research interests have different perspectives on the processes that assign subjective, affective meaning to stimuli. Some take a more cognitive stance in examining relatively stable affective phenomena, such as, for example, attitudes (Cunningham et al., 2007). Others focus more on the physiological and phenomenal aspects of affect and emotion and the temporary nature of emotional phenomena (Scherer and Moors, 2019). Evaluative priming research falls on the more cognitive side, as will become clear shortly. Thus, one might ask in what sense, if any, is evaluative priming related to affect and emotion? One general response is that emotion and cognition are deeply intertwined (see, e.g., Moors, 2007; Storbeck and Clore, 2007; Barrett, 2014), and evaluative priming research can thus, indeed, answer questions related to emotion and affect: When we extract information from our surroundings, translating it into “meaning,” we do so based on cognitive processes. This process of meaning making implies that we do not take the sensory input as it is, but we translate it into something. As Moors (2007, p. 1238) puts it: cognitive processes are those that “mediate between variable input-output relations by means of representations.” She proposes that “the unique feature of emotion has to do with the content of the representations involved in the transition from stimulus input to emotion.” This statement makes the relation of emotion to cognition as well as the importance of language for this relation very clear: Emotional information–as any other type of information–is processed *via* extracting some sort of category^2^. and mapping it onto a mental (potentially modal, embodied) representation, but the type of mental representation or the extracted information encompasses aspects specific to emotion and affect. Language, in turn, can be used to label the representation, and the representation might be partly or completely semantic in nature.

Two further distinctions are worth making, especially with regard to linguistic stimuli: the first is the one between *denotative* and *connotative* meaning (e.g., Osgood et al., 1957). The *denotative* meaning refers to the semantic mental representation of a word: For example, when reading the word HOUSE, a semantic mental representation is activated, which refers to the real object being referred to. The denotative meaning is identical or similar across individuals and the basis for communicating about the referred-to objects. *Connotative* aspects, by contrast, refer to all subjective, affective, or emotional associations of a stimulus (e.g., one could think of a warm and cozy home when reading HOUSE, or an old, gray, run-down tenement). These associations depend on an individual's stored mental representations created across their lifespan; thus, connotative aspects are multidimensional, and their content varies strongly between individuals (Osgood et al., 1957). Thus, when studying evaluation using priming paradigms, it is often the connotative aspect of the stimuli that is of interest. Sometimes, however, this distinction is not considered (e.g., when using emotional facial expressions, researchers often focus on their denotative meaning as positive or negative or representing a specific emotion). The second important distinction is the one between *affective* and *semantic valence* (Itkes and Kron, 2019). Itkes and Kron correctly pointed out that researchers often do not sufficiently differentiate between affective phenomena that are part of an emotional response–in the sense that they involve a physiological response or a potentially fleeting change in emotional feeling (i.e., affective valence, “hot” affect) –and affective phenomena that merely involve activation of factual knowledge (i.e., memory representations) of the valence associated with an object or event (i.e., semantic valence, “cold” affect). This distinction is important for evaluative priming, as there is ongoing debate about what exactly is activated in this paradigm and what mechanisms underlie the observed effects. We will come back to this issue later on.

Studying valence evaluation with priming paradigms is, therefore, a research field at the intersection of affect, cognition, and language: It uses predominantly linguistic stimuli (but also facial expressions, pictures, sounds) to study the automatic (i.e., fast, efficient, non-conscious, goal-independent, uncontrollable; Moors and De Houwer, 2006) assessment of valence, including the underlying processes, processing steps, or mechanisms, and mental representations.

## Is semantic or Affective Valence Activated in Evaluative Priming?

A core question in this area of research has been whether the affective connotation of words (and other stimuli) renders evaluative priming different from other forms of semantic processing, and whether the underlying processes or the activated mental representations differ from other semantic processes and representations. More specifically, researchers are interested in whether evaluative priming involves “hot” affective aspects (i.e., affective valence in terms of Itkes and Kron, 2019) –that is, activation of emotion components, such as facial expressions or physiological changes. We will now summarize the insights that empirical research has provided so far.

The question of whether evaluative priming differs from other forms of semantic categorization has been posed by this research field since its beginning (e.g., Fazio et al., 1986; Klauer and Musch, 2003; Storbeck and Robinson, 2004); however, only few empirical studies have directly studied this issue. One focus has been on whether evaluative categorization is more obligatory than semantic categorization and whether it occurs before or independently from semantic analysis (e.g., Zajonc, 1980; Nummenmaa et al., 2010). While many studies have investigated this question using categorization tasks without primes (e.g., Nummenmaa et al., 2010; Lai et al., 2012), some have employed an evaluative priming paradigm^3^ These early studies, by and large, showed comparable results: If evaluative categorization was the task at hand, evaluative priming effects emerged; if semantic categorization (e.g., person vs. animal) was the task at hand, semantic priming effects emerged (Kemp-Wheeler and Hill, 1992; Klinger et al., 2000; De Houwer and Randell, 2002; De Houwer et al., 2002), with no evidence for the additional activation of valence (see also Section How Is Valence Represented in Conceptual Representation Systems?). There is, however, one notable exception: Storbeck and Robinson (2004) systematically compared semantic categorization to evaluative categorization using the same stimuli, which varied not only with regard to valence but also with regard to category membership (i.e., animals vs. texture-related concepts vs. religious concepts; words in Exp. 1-3, pictures in Exp. 4-5). In four experiments, category congruency effects emerged and no evaluative priming effects, regardless of whether the task was semantic or evaluative categorization. Evaluative priming effects emerged only in one experiment, in which there was no variation of semantic category (Exp. 3). The authors explained this somewhat surprising finding with the salient variation in semantic category membership. According to them, evaluative priming effects only emerge when there is no (close) semantic relationship between primes and targets (but see Section How Is Valence Represented in Conceptual Representation Systems?), or when there is little semantic variation in a given task context. This already pointed to the conditionality of evaluative priming effects, an aspect that has been more systematically investigated in recent years and which we will elaborate on below. Of importance for the affective vs. semantic valence issue here is that there is evidence that evaluative categorization does not occur earlier than or independently of semantic categorization, but that semantic categorization seems to be the default processing operation when encountering (a wide range of) stimuli. Evaluative priming effects occur only when evaluation is important in the given context (e.g., due to the task set or attentional focus; see below), which is at odds with affective primacy.

Notwithstanding this documented predominance of semantic processes in the paradigm, one can ask whether a “hot” affect-related response (i.e., a physiological affective response or a partial reinstatement of such, as assumed by embodiment perspectives) is triggered by the prime, in addition to some semantic activation, and whether an affective state can influence evaluative priming effects.

With regard to the latter question, there are some hints that an affective state influences the effects: Foroni and Semin (2012) showed that inhibition of the zygomaticus major muscle–the muscle that pulls the lip corners apart in smiling–prevents evaluative priming effects. They argued that this is due to the involvement of affect-related, embodied processes in the paradigm. An alternative explanation would be that the muscle inhibition reduces positive affect. In support of this, Storbeck and Clore (2008) showed that participants in a neutral or happy mood showed evaluative priming effects but participants in a sad mood did not. These authors argued that affect influences the accessibility and use of semantic associations in memory, and that negative affect can inhibit activation spread in semantic networks. Thus, the observed effect would be the result of an affect-cognition interaction, and not evidence for the involvement of affect in evaluative priming *per se*. In a similar vein, Lemonnier and Alexopoulos (2019) examined the modulation of the evaluative priming effect by brief phasic affect, that is, trial-by-trial affective variations induced by positive and negative music or the manipulation of proprioceptive facial feedback *via* different facial muscle postures. Conceptually, they replicated Storbeck and Clore's (2008) results: Evaluative priming was observed in the neutral and positive conditions, but not in the negative affect conditions (also see Topolinski and Deutsch, 2013, for an influence of phasic affect on semantic priming). Consistent with this, De Saedeleer and Pourtois (2016; Exp.1) found that trait worry weakens the evaluative priming effect. Taken together, this line of research clearly shows that “hot” affect can influence evaluative priming effects; however, it seems unlikely that the processes underlying evaluative priming themselves encompass “hot” affect-related aspects, even though the available evidence cannot rule this out.

Another approach to the topic is the investigation of arousal influences on evaluative priming. Arousal is defined as the degree of physiological activation, and it is considered the second important dimension of the (semantic) affective space (Osgood et al., 1957; Russell and Barrett, 1999). Thus, if arousal affected evaluative priming effects, “hot” affect-related aspects might play a role in the paradigm. In three studies, Herring et al. (2015) used affective images rated low vs. high on arousal to examine whether prime arousal has an influence on evaluative priming effects. In their studies, greater prime arousal increased evaluative priming if primes were presented parafoveally, but not when primes were presented foveally. The authors argued this effect resulted from greater competition between sensory and response processes for high arousing parafoveal stimuli, which slowed down target processing in incongruent trials. Taking a more general perspective, the effects can be explained with arousal-biased competition theory (Mather and Sutherland, 2011), which proposes that arousal increases activation of salient or relevant stimuli and dampens the activation of less salient competitors. Consequently, the effects of Herring et al. can be explained by arousal-induced changes in attention and early vision, which are of particular relevance for parafoveal processing. If attentional processes are of less importance, as for example, in central vision, then arousal does not seem to play a role for evaluative priming. In line with this, Hinojosa et al. (2009) found that arousal did not influence reaction times in evaluative priming, although event-related potentials showed clear processing of the arousal aspect. However, Zhang et al. (2012) did observe a behavioral influence of valence and arousal: Using picture primes and word targets, they found that valence congruency effects were enhanced by high prime arousal. They also reported an effect of arousal congruency, which, however, was not mirrored in the corresponding event-related potentials. Also, as they used Chinese words (i.e., words of a logographic language), it is unclear whether the observed effects generalize to phonographic languages as well. So far, the very few existing studies suggest that arousal can impact evaluative priming effects; however, this influence seems to arise from arousal-induced influences on attention and downstream processing, not from the involvement of “hot” affect-related aspects in evaluative priming.

Thus, taken together, the existing evidence speaks against an involvement of affective valence in the evaluative priming paradigm. However, it remains a possibility that some “hot” aspects can be involved in the paradigm–as suggested by embodiment perspectives (e.g., Niedenthal et al., 2003), –and that research simply has not yet found the right approach to capture this influence (e.g., see Itkes et al., 2017, for an intriguing test of affective vs. semantic valence in the affective Simon paradigm).

## How is Valence Represented in Conceptual Representation Systems?

### The Evaluative Priming Paradigm and Its Mechanisms

Given the evidence presented above, it seems obvious that some form of semantic mental representation becomes activated in evaluative priming paradigms. But how is the activated valence mentally represented? This question cannot be answered without a close look at the mechanisms involved in the paradigm: Evaluative priming with the evaluative decision task is a variant of response interference tasks, like Stroop or flanker tasks (see Klauer et al., 1997; Wentura, 1999; also Wentura and Degner, 2010b). In the classical setup, target stimuli (e.g., words, pictures, faces, sounds) are categorized according to their valence (i.e., as positive or negative). The target (e.g., the word LOVE) is preceded by a briefly presented prime stimulus (e.g., the word BIRTHDAY) whose valence is either congruent to the target's response category or not. Typically, faster responses and/or fewer errors are observed in cases of congruency, which is taken as an indication of involuntary evaluation of the prime; that is, it is assumed that the valence of the task-irrelevant prime stimulus is automatically assessed. Thus, the paradigm is a parsimonious way to examine the automatic evaluation of the presented prime stimulus (in the example above: its connotative meaning) and the underlying processes and specific facilitatory conditions; it can potentially also reveal something about affect-cognition interactions. Moreover, given the task's setup, the question under investigation can be reframed as “Is the prime (distractor) compatible or incompatible with the response required by the target?” From this perspective, the most parsimonious explanation of the observed effects is based on response compatibility, that is, the assumption that the prime can facilitate either the correct or the incorrect response, with ensuing effects on response times [stimulus-response (or S-R)-based evaluative priming; see De Houwer, 2003]. Thus, evaluative priming can be conceptualized as just another variant of semantic categorization with response priming as the underlying mechanism [e.g., see Dehaene et al., 1998; Kunde et al., 2003; Rohr and Wagner, 2020 for studies with numbers; Klinger et al., 2000; Kiesel et al., 2006 for studies using other semantic categories]. Put differently: In the evaluative categorization task, stimulus valence is processed because it is response relevant. Thus, valence activated by a prime could just be seen as any other (response-relevant) semantic category. As such, a priming effect reveals (goal-dependent) involuntary activation of the prime's valence, but it does not reveal anything about the links between positive/negative concepts in semantic memory, for example, and thus provides little information about how valence is mentally represented.

However, the evaluative priming paradigm can be turned into a variant of the associative/semantic priming paradigm by changing task instructions (e.g., see Neely, 1991; McNamara, 2005; Wentura and Degner, 2010b). Concretely, if the task is to decide whether a target is a word or non-word, or to simply pronounce the target as quickly as possible, then response compatibility can no longer be the underlying mechanism. For example, a pronunciation task will mix valence-congruent prime/target pairs (e.g., *FLOWER-CAKE*) and valence-incongruent recombinations of prime/target pairs (e.g., *WAR*-*SUNSHINE*). Any resulting priming effects cannot be explained easily by S-R-based processes, but explanation must take into account processes that focus on the mental representation of prime and target and how activation of the prime representation helps or hinders access to the target representation [i.e., stimulus-stimulus (or S-S)-based priming; De Houwer, 2003].

Thus, changing an evaluative priming task from evaluative judgment to, for example, pronunciation is *prima facie* associated with a change in the underlying mechanism from response interference to encoding facilitation. This change brings up a very intriguing question: Will any prime render all stimuli of the same valence more accessible, such that a target of the same valence can be processed more quickly? Please note that this “encoding facilitation hypothesis” (Spruyt et al., 2002) with respect to valence as a concept differs from encoding facilitation for other semantic categories simply because (almost) every stimulus is assumed to have an evaluative connotation. Thus, the semantic “category” of positive/negative valence is much broader than other semantic categories. If this assumption holds, it suggests that valence is not only an important, easy-to-retrieve feature in the representation of an individual concept, but a powerful “connector” between representations.

We will now review the evidence in favor and against this encoding facilitation hypothesis, because – as mentioned earlier – this evidence allows for more insights into the underlying mental representations. That is, in the following paragraphs, we focus on evaluative priming evidence obtained with tasks other than the evaluative decision task. For a general overview, the reader is referred to Klauer and Musch (2003) and the meta-analysis of Herring et al. (2013). Having reviewed this evidence, we will outline the theoretical models that have been developed based on this research.

### The Encoding Facilitation Hypothesis

Early studies [Hermans et al. (1994, Exp. 2); Bargh et al. (1996), three experiments] provided initial evidence for evaluative priming with the pronunciation task, and thus for the assumption that evaluative concepts might facilitate access to other concepts of the same valence. Further evidence was found by Duckworth et al. (2002) in two experiments and Hermans et al. (2001, Exp. 2). However, later studies failed to replicate these early findings. For example, Klauer and Musch (2001) conducted a total of five experiments, all with null results (see also Hermans et al., 1996, Exp. 8; De Houwer et al., 1998; De Houwer and Hermans, 1999; Spruyt et al., 2004). Notably, in six experiments, Glaser and Banaji (1999) even found robust *reversed* effects, that is, faster responses in incongruent trials than congruent ones (see also Maier et al., 2003; Berner and Maier, 2004). Thus, early research using the pronunciation task yielded inconsistent results and provided no clear picture on whether stimulus valence can be activated when it is not task relevant. Using semantic classification of non-valence categories [i.e., animal/person (Exp. 1), object/person (Exp. 2)], De Houwer and Randell (2002) failed to obtain evidence for evaluative priming. Similarly, Klauer and Musch (2002) (Exp. 1–4) used four different binary classifications orthogonal to valence; none of the experiments showed an evaluative priming effect. Likewise, Hermans et al. (1998), as well as Rothermund and Wentura (1998), found no results with a color-naming task (see Warren, 1972, 1974, for its successful use in semantic priming research). Hermans et al. (2002) and Wentura (1998, 2000) only found evidence of evaluative priming in the lexical decision (word/non-word) task. However, to anticipate, these results can be explained by a further mechanism, *viz*. affective matching (Klauer and Stern, 1992; also see Klauer and Musch, 2002), which we further explain below.

Thus, in short, after initial positive findings, early research was characterized by null findings by positive evidence that can be explained by an alternative theory (i.e., affective matching), and even by reversed findings. This suggested that stimulus valence is not a special categorical construct and that it is only activated if it is response relevant. However, in subsequent years, there were several attempts to save the encoding facilitation hypothesis. This research proceeded from different starting points to identify possible moderator variables.

One approach of testing the encoding facilitation hypothesis with more scrutiny started from the premise that standard tasks might only involve superficial processing of the prime concepts, but that deeper processing may be needed to reveal encoding facilitation. For example, Spruyt et al. (2002) argued that early evaluative priming studies with the pronunciation task failed because word stimuli are only processed by a lexical executive system, bypassing semantic analysis (as argued by Glaser and Glaser, 1989), whereas pictures have privileged access to the semantic system (Glaser, 1992). In three experiments, the authors systematically varied the type (i.e., picture vs. word) of primes and targets. They found replicable evaluative priming of naming responses when pictures were used as primes, but not when words were used as primes. Significant effects with picture primes were also found by Duckworth et al. (2002, Exp. 1) and Giner-Sorolla et al. (1999, Exp. 2). Spruyt and Hermans (2008) replicated the picture-based effect again; interestingly, they found the effect already (and only) in the first block of trials, that is, on first occurrence of each stimulus. This is not unimportant to mention, because, in semantic priming research, non-repetition of materials is seen as a precondition of attributing priming effects unequivocally to semantic long-term memory retrieval (see, e.g., McNamara, 2005). Moreover, the result could also be interpreted in terms of Itkes and Kron (2019) distinction between semantic and affective valence. In a study with the affective Simon paradigm (Itkes et al., 2017), they showed that effects based on affective valence fade with habituation, whereas effects based on semantic valence did not. Thus, pictorial scenes might trigger an affective response (as generally observed for such pictures; Bradley et al., 2001), and this affective response facilitates access to congruent concepts. However, Klauer et al. (2016) pointed out that the comparison of evaluatively congruent vs. incongruent pairings in the study by Spruyt and Hermans was confounded with semantic relationships (i.e., on average, congruent prime-target pairs were semantically more related beyond valence congruency than incongruent pairs). In a preregistered replication study that avoided this confound, Klauer et al. found no significant evaluative priming effect. De Houwer et al. (2001) used word-word pairs in a pronunciation task with degraded (e.g., % U% G% L% Y%) and non-degraded targets. They argued that degraded presentation increased semantic processing of the word; hence, a potential facilitation by evaluative congruency might be more impactful. Indeed, an evaluative priming effect was found for degraded targets only. In a similar vein, De Houwer and Randell (2004) conducted two experiments with a conditional pronunciation task. That is, experimental trials were embedded in a sequence of filler trials with targets of a different category than the experimental targets (i.e., non-words, Exp. 1; occupation names, Exp. 2). The participants were instructed to pronounce the targets except when stimuli were non-words or occupations, respectively, that is, they were forced to semantically process the targets. The authors found significant evaluative priming effects in both experiments; however, they acknowledged that their effects may not reflect encoding facilitation, as the participants may have relied on a backward checking mechanism (Neely et al., 1989): Detecting affective congruency between prime and target was a valid indication of trial type (i.e., predictive of the decision “yes, target has to be pronounced”) since all filler targets were neutral. Thus, the obtained congruency effect might reflect a faster decision to pronounce the target in case of congruent pairs rather than indicate greater accessibility of the target due to the overlap in valence. (De Houwer and Randell, 2002, Exp. 2) found an evaluative priming effect (with word-word pairs) in a pronunciation task if the participants were instructed “to pay attention to the first word.” Thus, taken together, pronunciation tasks using pictorial primes or enforcing deeper encoding of target and/or prime yielded some replicable evidence of evaluative priming. Because of the many unclear and null results, however, this research has not provided a comprehensive explanation of S-S-based priming effects.

### Dimensional Attention and Goal Relevance

A more fundamental hypothesis about the validity and constraints of the encoding facilitation hypothesis has been proposed by Spruyt et al. in the last decade. The basic assumption is that “the semantic analysis of task-irrelevant stimuli is modulated by feature-specific attention allocation” (Spruyt et al., 2009, p. 37); that is, evaluative features are assumed to be processed only if attention is directed toward the evaluative dimension (see Kiefer and Martens, 2010, for a comparable assumption in semantic priming research). In three experiments, Spruyt et al. (2009) used a task-switching design to test their assumption. We will focus on Experiment 3 (which resolved some problems with the first two experiments): Depending on a cue (i.e., presence or absence of a green rectangle around the target), the participants had to pronounce target words or categorize them. Categorization trials constituted the majority (75%) of trials. In one group, the participants had to evaluate the targets in the categorization trials (i.e., label them as “positive” or “negative”); thus, the attentional focus was on evaluative features, even–according to the authors–in pronunciation trials. In the second group, the participants had to categorize the targets as objects or people; thus, the attentional focus was on semantic animacy features, even in pronunciation trials. Importantly, prime-targets pairs varied according to evaluative and semantic (i.e., object vs. person) congruency. The pattern of priming effects was in line with hypotheses: In the evaluative focus condition, pronunciation trials produced an evaluative priming effect but no semantic priming effect; the pattern was reversed for the semantic focus condition. Spruyt et al. (2012) replicated this evaluative priming effect even with masked primes, whereas Becker et al. (2016) reported two replication failures. A meta-analysis across all five experiments yielded a significant evaluative priming effect of *d* = 0.39.

Spruyt et al. (2007, Exp. 1) used the same basic design as Spruyt et al. (2012) but with an animal/object categorization task instead of pronunciation and pictures as both primes and targets. Again, a significant evaluative priming effect emerged in the non-evaluative trials (i.e., animal/object categorization trials) if the majority of trials were evaluative (i.e., valence-based categorization trials); the effect was not found if only a minority of trials was evaluative. Experiment 2 used words as primes and targets, and all trials required non-evaluative categorization (living vs. non-living); in addition, the participants were instructed to count the number of positive and negative primes to direct attention to the evaluative dimension. Again, a significant evaluative priming effect was found. In the same vein, Everaert et al. (2011) found an evaluative priming effect (in experimental trials) with pictures as primes and words as targets if filler trials (which constituted the majority of trials) used valent stimuli as well, but not if filler trials used neutral stimuli. An independent conceptual replication of Spruyt et al. (2007, Exp. 1) was published by Kawakami and Miura (2019), who used a mouse-tracking procedure (i.e., the participants categorized items by moving the mouse cursor to a response button). The evaluative priming effect found in non-evaluative trials was impressively large. However, a general critique of these task-switching paradigms (with a majority/minority of evaluation trials) is that the task set of the majority trials might carry over to the minority trials, thereby producing congruency effects. Consequently, the goal-dependent nature of evaluative priming effects, which requires response relevance (at least in part of the task), still holds for these tasks.

Therefore, Werner and Rothermund (2013) introduced a further paradigm that ensures attention is focused on the evaluative dimension without requiring evaluative decisions (or task switching). This paradigm includes trials with evaluatively neutral targets in addition to standard trials (with the variation of prime and target valence). Participants are asked to categorize targets as either valent (i.e., positive or negative) or neutral; thus, attention is on the evaluative dimension without valence discrimination (i.e., positive vs. negative) itself being response relevant. Importantly, the crucial congruency of prime and target valence is irrelevant for the response, which is identical for all valent targets; thus, explanations of an evaluative priming effect in terms of response facilitation or interference are ruled out. While the two experiments in Werner and Rothermund's study yielded null results, their research inspired a remarkable debate about the intricacies of such experiments (Rothermund and Werner, 2014; Spruyt, 2014), but it would be going too far to describe it here. Spruyt and Tibboel (2015) pointed out that some parameters (e.g., the inter-trial interval) used by Werner and Rothermund (2013) were non-standard; replicating their experiment with standard parameters yielded a significant evaluative congruency effect. However, Werner et al. (2018) emphasized a potentially important difference between their original experiment and the replication by Spruyt and Tibboel (2015): Whereas, the former authors used a fixed assignment of primes to targets to avoid any semantic or associative relationships (beyond valence congruency), the latter authors opted for random assignment. This solution entails the risk of a confound of evaluative congruency with semantic/associative relationship. Controlling for semantic associations, Werner et al. did not find an evaluative congruency effect.

In line with this emerging view that the goal relevance of the evaluative dimension is important was a finding by Gast et al. (2014), who introduced a new paradigm to test the encoding facilitation hypothesis. The authors proposed that resolving an evaluative mismatch of prime and target consumes cognitive processing resources, such that a mismatch distracts from *any* task that participants concurrently engage with (see Hermans et al., 1998, for an earlier suggestion of this idea). Gast et al. presented a sequence of two valent images, and a letter (X or Y) that was superimposed on the latter image had to be categorized. In two experiments, letter categorization was slower if pictures were affectively incongruent, but only if a secondary task set an evaluative context.

Spruyt et al. (2018) added a further twist to the discussion: They used a pronunciation task with word targets and picture primes. Pronunciation trials were mixed with a minority of so-called goal-induction trials that were identifiable by the letters “E” or “F” presented, instead of a target word. The participants were instructed and trained to categorize the letter in these trials *via* keypress, but only if one of four specific pictures preceded the letter. Thus, a small set of two positive and two negative pictures – randomly selected for each participant – acquired “goal relevance.” Remarkably, an evaluative priming effect was only found for trials with goal-relevant pictures as primes. Moreover, in line with Spruyt and Hermans (2008), the effect was restricted to the first block.

Taken together, these studies suggest that attention and some level of response relevance of the evaluative dimension are prerequisites for evaluative priming effects to emerge. Without response relevance, positive findings are more likely to be based on semantic/associative relatedness or – in the case of the Gast et al. study – are potentially based on a conflict reduction process (see also below). Thus, stimulus valence does not seem to cause activation spread to all concepts of the same valence. An exception might be affective pictures in studies that only yielded effects in the first blocks; such effects might be based on an affective response (Itkes et al., 2017). However, there has not been a systematic investigation of this assumption in the evaluative priming literature. A remarkable new line of research might be constituted by the study of Spruyt et al. (2018), who found evaluative priming effects only for those stimuli that had general goal relevance. Implications for models of the mental representation of stimulus valence are discussed further below.

### Mutual Facilitation

A different perspective on the underlying activated representations was taken by Wentura et al. (Wentura and Rothermund, 2003; Wentura and Frings, 2008; Schmitz and Wentura, 2012; Schmitz et al., 2014). They assumed that encoding facilitation might be a bidirectional process. Specifically, a prime might facilitate processing of a congruent target; in addition, however, a target might facilitate access to a congruent prime as well. Thus, it is assumed that both representations are activated in parallel. Testing this idea becomes interesting when prime and target are associated with the same valence, but different response tendencies. In this case, we might expect *reversed* priming effects (e.g., see Glaser and Banaji, 1999) because two concepts that mutually facilitate each other (because of the shared valence) compete for response determination. Wentura and Frings (2008) found evidence for this by using a picture/picture version of the priming paradigm and a naming task. In this version of the paradigm, target naming must be trained before the priming phase to achieve fast and unequivocal naming. Wentura and Frings added the primes to this training phase as well, with half of them trained with the adequate object name and half with a random response (i.e., consecutive numbers). Thus, primes and targets were presented before the priming phase, and object labeling was trained (i.e., the correct response was learned) or untrained for the primes. Interestingly, the untrained primes caused a positive priming effect, whereas the effect numerically reversed for the trained, response-bound primes. By changing the stimulus onset asynchrony to −80 ms (i.e., the prime was presented on top of the bigger target picture 80 ms after the target onset), Schmitz and Wentura (2012) found a symmetric pattern of a positive priming effect for untrained primes and a significant reversed effect for trained primes, indeed indicating that prime and target might be activated in parallel. This parallel activation is difficult to explain with encoding facilitation, which assumes that activation spreads from one concept to others (such that activation dissipates for the prime if the target becomes activated). Schmitz and Wentura corroborated this pattern of results with a semantic classification task (i.e., persons vs. animals) using pictures (Exp. 2) or words (Exp. 3; also see Schmitz et al., 2014). Interestingly, the authors found that the to-be-expected *semantic* congruency effect was moderated by *evaluative* congruency, which implies that stimulus valence was processed despite being response irrelevant. These effects can only be explained with mutual facilitation of prime and target.

In sum, the available evidence paints a rather complex picture. Evaluative priming effects with the evaluative categorization task are a robust phenomenon, which can, however, be explained with response compatibility as the underlying mechanism. This makes it difficult to gain much insight into the underling mental representation of valence and the links between concepts. Evidence accrued with non-evaluative categorization, pronunciation, and lexical decision tasks sheds more light on the underlying representations – however, the results are complicated: With some caution, we can state that there seem to be conditions that yield evaluative congruency effects with these tasks. Specifically, attention or goal relevance and sufficiently deep (i.e., semantic) processing of the evaluative dimension seem to be required for such effects to emerge. Moreover, at least in two cases, evaluative congruency effects were found to depend on additional semantic associations between prime and target (Klauer et al., 2016, in a replication of Spruyt and Hermans, 2008; Werner et al., 2018, in a replication of Spruyt and Tibboel, 2015). Thus, associative relationships at least boost the evaluative priming effects. Given this overall, how can the mental representation of valence be modeled?

### Theoretical Models of S-S-Based Evaluative Priming

For a long time, the guiding hypothesis of S-S-based evaluative priming was encoding facilitation of the target concept by an evaluatively congruent prime *in long-term semantic memory*.

This encoding facilitation was first explained with the theory of semantic networks (Anderson, 1983; Collins and Loftus, 1975; for a recent discussion, see Kumar et al., 2021): Semantic concepts are assumed to be represented by nodes in a network; links between nodes symbolize semantic or associative relationship. Processing a word leads to the “activation” of the corresponding node and a process of “spreading activation” that increases the activation level of related concepts *via* the links. Hence, these concepts become more accessible for processing compared to a baseline condition. The semantic network theory was adapted by Bower (1991) to explain mood congruity effects and was also embraced by evaluative priming researchers. The guiding assumption is that a “positivity node” and a “negativity node” exist, linking all positively and negatively connoted concepts, respectively. However, it can be argued that a semantic network will be functional only if the amount of spreading activation is constrained by a reasonable number of links emanating from the source node – otherwise, activation (e.g., from a generic positivity or negativity node) of target concepts will become too small to be measurable (a “fan effect”; see Anderson, 1974; also see Hermans et al., 1996; Wentura, 1999; Spruyt et al., 2002). There are several additional problems: First, semantic priming experiments use unique primes and targets to unequivocally interpret observed effects in terms of semantic long-term memory retrieval (see, e.g., McNamara, 2005); by contrast, most evaluative priming experiments use comparably small prime and target sets with a high repetition rate (for exceptions, see Klauer and Musch (2001), Exp.1; Spruyt and Hermans (2008). Second, when comparing evaluative and semantic priming effects, one should keep in mind that evaluative priming can be considered a special case of semantic priming, that is, priming of category members by category coordinates that are not (necessarily) associated (e.g., *robin-eagle* as exemplars for the category *bird*). For semantic priming, Hutchison (2003, p. 804) summarized that “the overall evidence of [semantic] priming from category coordinates is weak” (but see Lucas, 2000). If this conclusion holds, the *a priori* expectation of finding S-S-based evaluative priming effects is rather low. Or in other words: If there is some evidence for S-S-based evaluative priming effects, we should carefully discuss whether such effects should be interpreted as semantic priming effects because, in general, evidence of semantic priming from category coordinates is weak. Semantic network theory should thus be seen more as a *metaphor* that illustrates the phenomenon of priming effects, but it does not really explain the phenomenon in a deeper sense.

More promising (at least at first glance) are parallel-distributed models of priming (e.g., Masson, 1995; McRae et al., 1997). In these models, each semantic concept corresponds to a specific pattern of activated processing units, and the semantic relatedness between concepts is determined by the number of shared activated units. This conceptualization is more far-reaching than that of semantic networks, since parallel-distributed models contain assumptions about the emergence of the structures (*via* learning mechanisms). Priming effects are explained by a faster transition from the pattern representing the prime concept to a semantically related one (i.e., the pattern representing the related target) than to an unrelated pattern because the shared units are already in the appropriate mode of activation. Although this model was developed to explain semantic priming (see McNamara, 2005), it is prima facie perfectly suited to account for S-S-based evaluative priming as well, under the assumption that a considerable part of the activation pattern represents the evaluative features of a concept (see Wentura, 1999, 2000; Spruyt et al., 2002).

However, there are two main problems associated with this hypothesis: First, Spruyt et al. (2007) pointed out that, according to simulation studies by Masson (1995), the overlap of prime and target representations must be very large to account for priming effects; this would mean that the evaluative part of the stimulus representation must be an implausibly large part of the representation of concepts. The second problem of parallel-distributed models of priming was discussed by Schmitz and Wentura (2012). They argued that some findings in evaluative priming research can only be explained by parallel activation of prime and target representations (e.g., reversed priming effects with the pronunciation task; Glaser and Banaji, 1999; Wentura and Frings, 2008; Schmitz and Wentura, 2012). As parallel-distributed models assume that there is a transition from the prime pattern to the target pattern (which is faster if concepts are related), the prime representation should no longer be accessible after this transition. Scherer and Wentura (2018) directly tested the assumption of parallel activation using a post-cue priming task (i.e., a cue following a brief and masked presentation of two words indicates which of the two words has to be identified). Results were in line with the assumption of parallel activation, suggesting that parallel-distributed models might also not be suitable to explain the activations underlying evaluative priming effects.

Thus, in a nutshell, S-S-based evaluative priming effects are not (easily) explainable with the two well-known long-term memory models that were used to explain semantic priming effects. So how can we explain them? In our view, three mechanisms need to be considered to better understand the effects. In our view, these mechanisms (and the activated prime and target representations) have to be modeled as operating in working memory, not in long-term semantic memory.

We already mentioned *affective matching theory* by Klauer and Stern (1992): According to this theory, if two evaluative stimuli are presented, the two valences are (a) involuntarily processed and (b) involuntarily compared; this comparison results in an affective match or mismatch. Depending on the context, match or mismatch influences ongoing behaviors, primarily *via* affirmative or negative response tendencies. Since binary decision tasks often involve affirmation (e.g., “yes, this is a word”), congruency effects (i.e., faster responses in the match case) are predicted. However, the touchstone of such a theory is the case of negation. If a task set can be changed to a negation situation *via* instructions (e.g., in a lexical decision: “no, this is *not* a senseless letter string”), the effect should reverse. This was, indeed, observed in some studies (see Klauer and Stern, 1992; Wentura, 1998, 2000; Klauer and Musch, 2002; Rothermund and Werner, 2014). Affective matching mechanisms are especially likely to govern the effects if *pairs* of stimuli have to be compared on a feature that is orthogonal to valence [e.g., do the words share lettercase (lower or uppercase)?]. Klauer and Musch (2002) tested this in four experiments with different aspects, and found results that were in line with matching theory: If the “same” response was (by instruction) affirmative, it was facilitated by the task-irrelevant evaluative congruency of the stimuli (compared to incongruency); if the “different” response was affirmative, the effect was eliminated and tended to be reversed. Note that the matching mechanism is about the comparison of two activated concepts; hence, an obvious suggestion is to locate it in working memory. However, effects found with the pronunciation task are problematic for this theory because pronouncing a word is not a judgment of affirmation or negation.

Such effects might be explained by a further mechanism, that is, *affective motivational conflict reduction*. Specifically, Hermans et al. (1998) suggested that the affective incongruency of two stimuli *per se* slows down system behavior due to an inherent processing conflict. They argued that automatic evaluation results in action tendencies of approach (for positive stimuli) or avoidance (for negative stimuli). In case of a mismatch, the action tendencies are in conflict; this conflict blocks behavior until it is resolved. However, it might be sufficient to assume that an affective match or a mismatch serves as a signal: A match might signal: “everything as expected; go on!”, whereas a mismatch might signal: “violation of expectation; clarify!” However, in four experiments with a color-naming task with evaluatively congruent or incongruent prime-target pairs, Hermans et al. did not find evidence for their claim (see also Rothermund and Wentura, 1998). The evidence presented by Gast et al. (2014; see above) on the other hand fits the affective-motivational account. This theory is, in principle, able to explain congruency effects in all evaluative priming tasks (although it does struggle to explain reversed effects). Its strength, however, lies in the potential to explain effects if *both* valent stimuli are *task irrelevant* (in contrast to affective matching theory, which is best suited to explain effects if both stimuli are task relevant). Again, the conflict reduction account is about the comparison of two activated concepts; hence, again, an obvious suggestion is to locate it in working memory.

A third candidate account relates to processes based on *mutual facilitation or inhibition in working memory*. Wentura et al. (Wentura and Rothermund, 2003; Wentura and Frings, 2008; Schmitz and Wentura, 2012; Schmitz et al., 2014) emphasized the parallel activation of prime and target concepts and their mutual facilitation and/or inhibition (see Section Mutual facilitation above). Depending on task context, parallel activation can have different consequences (Wentura and Rothermund, 2003). Task context can be characterized by two dichotomies: The first dichotomy refers to whether participants have to categorize the valence (in an evaluation task, or, more generally, a response priming design) or not (in tasks such as pronunciation or a lexical decision, or, more generally, a semantic priming design). The second dichotomy refers to whether or not the prime serves as a distractor competing with the target for determination of the response.

To elaborate on the latter: In the evaluation task, the usual instruction to respond fast might lead to the following processing strategy: “Whenever the stimulus display delivers enough evidence for a positive or negative response, I will press the corresponding key. “One might add: ”… whatever the basis for this evaluation might be.” That is, participants may not try to separate prime and target (also see the evaluation window account; Klauer et al., 2009). Thus, congruent pairs will facilitate the response because the evidence provided by the stimulus configuration is unambiguous. If, however, the participants follow the strategy to base their decisions on the correct source of evidence only – that is, on target valence – the prime is, in fact, a distractor whose activation hinders correct response formation. Thus, in the case of congruency, the competition between prime and target representations is prolonged by the congruent target facilitating processing of the prime and/or in the case of incongruency, competition is shortened due to the incongruent target inhibiting processing of the prime. If the participants use this strategy, reversed effects (e.g., Wentura and Rothermund, 2003; also see Klauer et al., 2009,^4^) – or at least reduced congruency effects (see, e.g., Wentura and Degner, 2010a) – can be found even with the evaluation task.

The pronunciation task can serve as another example: In this task, participants obviously need to focus on the target representation to pronounce the target; thus, in a general sense, the prime is always a distractor. Response execution must, therefore, wait for the activation of the target representation to reach the threshold and activation of the prime representation to be diminished (Houghton and Tipper, 1994). This can be seen as a routine process, given that reading of words almost always occurs in the context of other words. However, in the priming task, we can create, more or less, competition by associating the distractor with automatic response tendencies or not (Wentura and Frings, 2008; Schmitz and Wentura, 2012). If there are no such competing response tendencies, we might find evidence for congruency effects, because a congruent prime helps to establish the activation of the target representation, especially with the usual prime-target onset asynchrony. If, however, the distractor is associated with a competing response tendency, the mutual facilitation in case of congruency can prolong the competition between a distractor and a target, especially with a negative onset asynchrony of prime and target (Schmitz and Wentura, 2012). Interestingly, a working memory model that was developed in a totally independent area of research – the dual-store neuro-computational model of short-term memory (Usher and Cohen, 1999; Haarmann and Usher, 2001; Davelaar et al., 2005, 2006) – proposes mutual facilitation processes based on semantic similarity in working memory. Given this backdrop, Scherer and Wentura (2022) used a change detection task – that is, a typical working memory task – with emotional (i.e., angry and happy) faces as stimuli to investigate the effects of evaluative congruency on working memory performance. They found an (admittedly: weak) effect of evaluative congruency, with better performance on trials with evaluatively congruent compared to incongruent displays. This effect is in line with the assumptions that S-S-based evaluative priming effects might arise from mutual facilitation/inhibition of simultaneously active evaluatively congruent concepts.

In Section Dimensional Attention and Goal Relevance, we emphasized in the concluding paragraph the recent study by Spruyt et al. (2018) who found evaluative priming in the pronunciation task only for those stimuli, which had general goal relevance: Concretely, a small set of two positive and two negative prime pictures indicated that, if a letter instead of a target stimulus will be presented in a given trial, it had to be categorized. (For all other primes, no response to the letter was allowed). We can interpret this finding in the following way: In contrast to goal-irrelevant primes, who might produce a very superficial activation of its mental representation, a goal-relevant prime might mandatorily become part of the current working memory to prepare for the potential letter classification task. Given this status, the primes might facilitate congruent targets.

Taken together, the three outlined mechanisms – as different as they might seem at first glance – have much in common: All three approaches are compatible with the assumption that two concepts are activated in parallel and that evaluative congruency vs. incongruency influences further processing while both concepts are activated. It then depends on specific task parameters which of the three mechanisms determines observed effects. That is, we can identify task contexts that produce results that are either more compatible with affective matching, conflict reduction, or mutual facilitation/inhibition. If both stimuli are task relevant, the affective matching process might be dominant; if both stimuli are task irrelevant, the conflict reduction process might be dominant. Finally, if only one stimulus is task relevant, mutual facilitation or inhibition might be the processes that determine evaluative congruency effects. Of course, there is overlap: The affective matching mechanisms might be evoked if only the target is task relevant: However, a clear judgmental context must be given that asks for affirmation or negation; then, affective match or mismatch meddles as well with giving a response (Wentura, 1998, 2000; Klauer and Musch, 2002 Exp. 9). The conflict reduction process might, in principle, be at work in all three task contexts. However, it cannot be the sole account because some results cannot be explained by it (e.g., reversed effects found with the pronunciation task, Schmitz and Wentura, 2012, or the effects found by Klauer and Musch (2002) see above, that fit to affective matching theory).

Finally, to (provisionally) answer the question posed at the outset of this section – “How is valence represented in conceptual representation systems?” – we can conclude that valence connotations are –in a specific sense – a fundamental part of the representation of semantic concepts: Evaluative information will be involuntarily activated in specific contexts, that is, if attention or goal relevance is given. Then, the evaluative aspects of the corresponding concept are accessed (which might be equated with: is activated in working memory). However, the “in a specific sense” constraint should be understood as: There is not much evidence for a conceptual representation system that has valence as a basic organizational feature such that processing of a positively connoted concept renders all other positive concepts more accessible, for example. But if context conditions trigger the activation of evaluative connotations and two concepts are activated in parallel (which might be equated with: both are concurrently represented in working memory), then affective match or mismatch will influence behavior.

## Can Further Emotion Aspects be Involuntarily Activated?

From what has been discussed so far, we might conclude that there is not much special about the processing of stimulus valence in evaluative priming paradigms. Semantic valence seems to be activated, and this happens only under specific processing conditions. Nevertheless, there is evidence suggesting that there could be special aspects in the processing of emotion and affect in evaluative priming. Recently, researchers have started investigating the processing of specific emotions or specific emotion aspects (i.e., possessor/other relevance, potency, specific emotion categories) with the paradigm. From the viewpoint of semantic processing accounts, one might think that the processing of such aspects occurs if they are task relevant and similar to a semantic category. The emerging evidence, however, tells a different story. For example, (Wentura and Degner, 2010a, also see Degner et al., 2007; Degner and Wentura, 2011) examined whether possessor vs. other relevance plays a role in evaluative priming effects. This differentiation was introduced by Peeters (1983) who posited that many trait adjectives are not simply positive or negative, but that the evaluation depends on the perspective of the evaluator. For example, being aggressive is not necessarily bad for the aggressor her/himself but is probably seen as bad by the social environment; that is, it is an other-relevant trait. Likewise, being intelligent is a positive trait for the trait holder, but not necessarily for others, so that this trait is (primarily) possessor relevant. While this distinction is interesting from a social viewpoint, one might assume that some controlled processing or conscious reflection is necessary to make it. On the contrary, however, the studies by Wentura et al. showed that this differentiation is of relevance in evaluative priming paradigms. For example, Wentura and Degner (2010a) observed masked evaluative priming effects in two studies only when prime and target were congruent with regard to *both* valence *and* possessor/other relevance. Of note, their analysis controlled for semantic relatedness, so the differentiation cannot simply be explained by the assumption that concepts of the same relevance type are more closely connected semantically. The authors thus proposed that relevance type is also assessed early and automatically with stimuli for which this differentiation is relevant. Several other studies corroborated this finding, also under masked presentation conditions (Degner et al., 2007; Degner and Wentura, 2011; De Paula Couto and Wentura, 2012).

While the possessor/other relevance differentiation certainly has some appeal, it can possibly be subsumed under a more general point: It might be the case that the specific emotional connotation is activated instead of coarse valence and that only prime-target pairs with matching connotation are processed as congruent (e.g., *incapable – depressed* might be perceived as congruent because both relate to sadness). To examine this hypothesis, Rohr et al. (2012) built on work by Carroll and Young (2006), who adapted the evaluative priming paradigm to specific emotions. In this paradigm, participants are given four response options and have to categorize targets according to the specific emotion category (i.e., joy, anger, fear, sadness). Carroll and Young (2006) found priming effects based on emotion-category congruency in four studies using words, facial expressions, and emotional sounds. However, they used an unusually long stimulus-onset asynchrony (SOA; i.e., the time from the prime onset to the target onset) in all but one experiment (i.e., 750 ms; 250 ms in Exp. 4), and analyzed data at a rather coarse level of granularity (i.e., comparing all emotion-congruent to all emotion-incongruent conditions; with the exception of Exp. 4, which excluded the positive emotion). Thus, results could not rule out a certain level of controlled processing, and the degree to which the participants differentiated emotions remained clear: Specifically, did the participants differentiate between all emotion categories? Or did they only differentiate specific emotion aspects, such as the abovementioned possessor/other relevance? Rohr and colleagues (Rohr et al., 2012; Rohr and Wentura, 2014; Wentura et al., 2017) examined the degree of processing differentiation in more detail using a masked version of the emotion priming paradigm. Their analysis focused on the prespecified set of Helmert contrasts: The first contrast treated all positive-positive and negative-negative (e.g., sadness-anger) prime-target combinations as congruent and all combinations of positive and negative valence as incongruent, thereby testing for differentiation of valence. The next contrast compared anger vs. fear/sadness combinations, excluding all trials containing the only positive emotion, joy. Specifically, anger/anger trials, as well as all combinations of fear/sadness, were treated as congruent, and all trials involving anger *and* fear or sadness as incongruent, with the rationale, that the two latter emotions both convey the same type of relevance (i.e., possessor relevance; alternatively, this differentiation can be interpreted as an assessment of potency, Neumann et al., 2020; or coping ability, Rohr et al., 2020). The last contrast compared the remaining two emotions (i.e., all trials involving fear/sadness only). Rohr et al. (2012) found emotion priming effects beyond valence, concretely a differentiation of anger vs. fear/sadness, but no differentiation of each specific emotion category (i.e., fear and sadness were not differentiated). The authors interpreted the finding as evidence for the extraction of valence and possessor/other relevance. What distinction exactly underlies this intermediate differentiation is a matter for debate; however, in any case, the finding is very interesting for the issue of underlying processes: From a semantic processing (and response compatibility) viewpoint, emotion-specific priming effects should have emerged. Thus, the observed intermediate differentiation cannot easily be explained by semantic processing accounts. By contrast, the findings provide preliminary evidence that early processing can extract more than just valence, for social and affect-related reasons (also see Rohr et al., 2020). Of note in this regard, different patterns of early differentiation beyond valence have been observed. In other studies (Rohr and Wentura, 2014; Rohr et al., 2015), an intermediate differentiation of anger/fear vs. sadness emerged, which can be interpreted as a differentiation along the arousal or threat dimension, albeit with a different paradigm (Rohr et al., 2015) or using specific stimuli (i.e., low spatial frequency-filtered facial expressions; Rohr and Wentura, 2014). This inconsistency leaves room for an interpretation along methodological lines: Possibly, individuals can only focus on two stimulus dimensions under fast, unintentional processing conditions such that only two response categories yield specific effects (please note that clearly visible primes yielded congruency effects for all four emotion categories in; Rohr and Wentura, 2014; Rohr et al., 2015).

Wentura and Rohr (2018) tackled this issue with a further version of evaluative priming – the “leave-one-out” paradigm. In four studies, the participants completed emotion priming tasks with only two target categories (e.g., anger vs. sadness); however, there were three categories of negative masked primes (i.e., anger, fear, sadness), with primes drawn from a different set than targets. This setup allows examination of the impact of a masked prime that is not part of the task set, for which, presumably, no response trigger is built up. In a nutshell, results supported an interpretation in terms of emotion-specific processing, however, with task-relevance playing a primary role: The response-relevant primes always yielded clear congruency effects, whereas priming effects were approximately halved with the “left-out” prime. This suggests that the left-out prime category is differentiated from the other two response-relevant categories, but that it does not lead to the activation of a “response trigger,” given that no response category is associated (see Kunde et al., 2003, for the action trigger account). These results mesh well with the results of Neumann and Lozo (2012), who observed emotion-specific priming effects for masked disgust and fear stimuli in three studies with a disgust vs. fear categorization task.

Recently, Neumann et al. (2020) have also adapted the evaluative priming paradigm to study the automatic processing of potency, which the authors defined as an umbrella term for power, dominance, control, and related physical concepts, such as weight and size. After valence and arousal, potency is a further dimension defining the affective semantic space (Osgood et al., 1957), but only few studies have targeted this dimension explicitly. In three studies, Neumann and colleagues showed that a potency categorization task (i.e., classify the target as strong vs. weak) reliably elicits potency priming effects, but only under specific conditions (i.e., with a short SOA, a response window procedure, with adjectives but not nouns). They also showed that the effects depend on task instructions: With a potency categorization task, no effects for valence (Exp. 1a; Exp. 3) or arousal (Exp. 1b) were observed even though the stimuli varied on these dimensions. In a valence categorization task (Exp. 3), the potency priming effect was reduced but still significant. The authors attributed this latter finding to the salience of this dimension in their stimulus pool. Importantly, the potency priming effects still held when controlling for semantic overlap, suggesting that the processing of this dimension is “an important mechanism in addition and beyond valence that regulates social behavior and prepares the organism to act adaptively” (Neumann et al., 2020, p.2).

Thus, taken together, studying emotion-related aspects or dimensions with priming paradigms shows that more than valence can be extracted early, and even under masked presentation conditions. Similar to the evaluative effects discussed earlier, the effects seem to depend on specific task parameters. However, the effects cannot be explained solely by semantic associations; whether certain aspects or dimensions are processed seems to be related to their social importance. Perhaps, the most interesting finding in this regard is that processing of specific emotions under short and masked presentation conditions seems to be confined to two dimensions only, even if the task requires processing of the specific emotion. Given the results of Wentura and Rohr (2018), this two-dimensional pattern might be related to task complexity (i.e., four-response options). Alternatively, it might reflect a more general restriction of processing: emotion research and social psychological research have often found two important dimensions (Russell and Barrett, 1999; Fiske et al., 2002; Oosterhof and Todorov, 2008), and recent research has also suggested that current goals moderate which dimensions or aspects are attended to (Nicolas et al., 2022). In this respect, the results might also align with the abovementioned working memory account of evaluative priming. Perhaps, only two dimensions are kept active in working memory under implicit processing conditions. However, more research is needed to improve our understanding of these matters.

## Summary and Conclusion

To summarize, research using evaluative priming paradigms has revealed that information in our environment is evaluated automatically; that is, prime valence is processed unintentionally, efficiently, and also non-consciously, if (and only if) the evaluative dimension is relevant to the task goal. Moreover, the underlying mental representations clearly seem to be semantic in nature. “Hot” processing does not seem to be involved. A few studies, however, give rise to the assumption that the affective state can influence priming effects (Storbeck and Clore, 2008; Foroni and Semin, 2012; Lemonnier and Alexopoulos, 2019). Furthermore, some studies with picture primes, which found effects in the first block only, leave open whether an affective response might contribute to evaluative priming effects in studies employing pictures; however, systematic research into this question is still lacking. Thus, despite three decades of research with the paradigm, the complex pattern of evidence still does not allow an authoritative answer to the question of how to model the mental representations and activated valence of prime and target. The proposed long-term memory models all struggle to explain the evidence in its entirety. We proposed a working memory account, which, however, requires further development.

Does this mean that there is nothing special about the processing of stimulus valence and that it should be equated to other semantic categories? We do not think so, even if the activated valence reflects factual knowledge (i.e., semantic valence). As described in Section Can Further Emotion Aspects be Involuntarily Activated?, observed effects seem related to the social or personal relevance of the information, and suggest that there is some early extraction of affect-related information beyond valence. It could be that this automatic processing is restricted to the processing of two socially relevant dimensions only, thereby converging with other social theories (e.g., Nicolas et al., 2022), but, again, further research is necessary to corroborate this notion.

Thus, to conclude, the evaluative priming paradigm has revealed much about the link between emotion and cognition: Semantic affective knowledge is automatically activated when encountering stimuli in a context in which the evaluative dimension is relevant. It is even possible that additional socially relevant emotion aspects are activated in this situation. Existing research suggests that the effects are not explainable with theories of semantic priming. In that sense, the processing of stimulus valence might still be special. How *exactly* it is special, however, has not been specified in a comprehensive theoretical account just yet.

## Author Contributions

MR and DW conceived the review and wrote substantial parts of the manuscript text. Both authors contributed to the article and approved the submitted version.

## Funding

We acknowledge support by the Deutsche Forschungsgemeinschaft (DFG, German Research Foundation) and Saarland University within the funding programme Open Access Publishing.

## Conflict of Interest

The authors declare that the research was conducted in the absence of any commercial or financial relationships that could be construed as a potential conflict of interest.

## Publisher's Note

All claims expressed in this article are solely those of the authors and do not necessarily represent those of their affiliated organizations, or those of the publisher, the editors and the reviewers. Any product that may be evaluated in this article, or claim that may be made by its manufacturer, is not guaranteed or endorsed by the publisher.

## Footnotes

^1^Of course, we do not assume that every mental concept has an existing evaluative connotation. When encountering a stimulus for the first time, evaluation and/or an affective response occur for the first time, and the response is constructed online. By and large, empirical research employing the evaluative priming paradigm has, however, focused on clearly evaluative (mostly normative) stimuli whose evaluative connotation can be assumed to be stored in memory.

^2^We use the term category here in a cognitive-science sense. As Harnad (2005) (p. 20) puts it: “We, organisms, are sensorimotor systems. Things in the world come in contact with our sensory surfaces, and we interact with them based on what that sensorimotor contact “affords.” All of our categories consist of ways we behave differently toward different *kinds* of things – things we do or do not eat, mate with, or flee from; or the things that we describe, through our language, as prime numbers, affordances, absolute discriminables, or truths.” From that viewpoint, emotions can be seen as one kind of category (see also Boster, 2005).

^3^We focus here on the evaluative categorization task with positive/negative primes and targets. There is also a very similar paradigm: the affect misattribution procedure (Payne et al., 2005; note this was, initially, also referred to as the affective priming paradigm, Murphy and Zajonc, 1993). Reviewing the evidence from this paradigm is beyond the scope of the present review. In a nutshell, research using this paradigm provides different answers to the questions outlined here; the underlying processes and mechanisms are different (see, e.g., Gawronski et al., 2010 see Payne and Lundberg, 2014, for an overview), as are the underlying activated representations (e.g., De Houwer and Tucker Smith, 2013; Rohr et al., 2015).

^4^Klauer et al. (2009) presented an alternative explanation for reversed effects in the evaluation task. We will not elaborate on this account because our focus in this review is on S-S-based evaluative priming (i.e., evaluative priming in the semantic priming design).
